# 

**Published:** 1890-06-21

**Authors:** 


SATURDAY, JUNE 21st, 1890.

**A Dangerous Hospital** - 161

**Words of Consolation.**—Redemption - 162

**The Doctor's Pulpit.**—Those that Travel by Water - 163

**Turning Over New Leaves.**—A City Girl - 164

**Before the Lords' Committee** (By our Special Commissioner). IV.—Mr. William Bousfield and Dr. W. B. Clarke 165

**Midwives of Old** - 166

**Everybody's Page** - 167

**Notes and News** - 168

**A Wage Limit for In-Patients** - 169

**Annotations.**—Mrs. Gladstone's Appeal—"Brain-fag" in Women—Jews and Athletics - 170

**Books Received** - 173

**The Practitioner's Mirror and Retrospect:**

Surgery—Skin Grafting - 171

Dietetics—Use of Alcohol - 171

Therapeutics—Administration of Santonin—Iodide of Calcium as an Antiseptic - 172

New Drugs, Appliances, and Things Medical—Messrs. Down Brothers' Surgical Instruments - 173

**The Hospital Workshop** (By Our Special Commissioner). XXVII.—The Hebrew Wards at the London Hospital - 174

**Working Women in Large Towns.** IV.—Class II. Factory and Workroom Hands (*continued*) - 175

**Passing Topics.**—"Stricter Control." - 176

**Scraps and Gleanings** - 176

### EXTRA SUPPLEMENT—THE NURSING MIRROR.

**En Passant.**—Short Items—Excursions—Our Infirmary Baby—Matron and House Surgeon - xlv
**Sketches of Insanity** (By Francis H. Walmsley, M.D.). I.—Introductory - xlvi
**The Nurse's Bookshelf.**—A History of District Nursing xlvi
**A Nurse in a Lodging House** - xlvii
**Examination Questions.**—Prize Answer for May - xlvii
**Everybody's Opinion.**—Fever Nurses - xlviii
**Appointments.**—Notes and Queries - xlviii
**The Fourth of July** - xlviii
**Vacancies** - xlviii
**Amusements and Relaxation** - xlviii

